# Fibroblast growth factor 23 (FGF23) gene polymorphism in children with Kawasaki syndrome (KS) and susceptibility to cardiac abnormalities

**DOI:** 10.1186/1824-7288-39-69

**Published:** 2013-10-29

**Authors:** Fernanda Falcini, Donato Rigante, Laura Masi, Marcello Covino, Francesco Franceschelli, Gigliola Leoncini, Giusyda Tarantino, Marco Matucci Cerinic, Maria Luisa Brandi

**Affiliations:** 1 Department of Internal Medicine, Section of Rheumatology, Transition Clinic, University of Florence, Florence, Italy; 2 Institute of Pediatrics, Università Cattolica Sacro Cuore, Rome, Italy; 3 Department of Surgery and Translational Medicine, University of Florence, Florence, Italy; 4 Department of Internal Medicine, Università Cattolica Sacro Cuore, Rome, Italy; 5 Department of BioMedicine, Section of Rheumatology, Transition Clinic, University of Florence, Viale Pieraccini 18, 50139 Florence, Italy

**Keywords:** Kawasaki syndrome, Fibroblast growth factor 23, Cardiac abnormalities, Child

## Abstract

**Background:**

Fibroblast Growth Factor (FGF) 23 influences endothelial integrity and few reports have studied the association between FGF23 and Kawasaki syndrome (KS), a childhood vasculitis displaying a high risk of subsequent cardiac abnormalities (CaA).

**Aim:**

To investigate the genetic variation in the *FGF23* gene in a cohort of KS children and its association with serum FGF23 levels and eventual development of CaA, including both coronary artery dilatations and aneurysms.

**Patients and methods:**

84 Italian KS children were recruited; 24/84 (28.6%) developed CaA. Each patient underwent evaluation of serum FGF23 levels and *FGF23* genotype: the frequency of the c.212-37insC (rs3832879) polymorphism in intron 1 was examined and compared with sex, age at disease onset, fever duration, laboratory data, and occurrence of CaA. Univariate statistical analysis of categorical parameters was performed by the Pearson’s Chi-square test or Fisher’s exact test as appropriate. Parametric variables were assessed by Student’s t-test for unpaired data. Independent predictors of disease were studied by a logistic regression model.

**Results:**

28/84 patients carried the *FGF23* polymorphism (33.3%) and had higher serum FGF23 levels (*p* < 0.01). *FGF23* polymorphism was significantly associated with CaA compared to wild type *FGF23* children (respectively, *p* = 0.03 and *p* = 0.05). The comparison with demographical, clinical or laboratory data was not significant.

**Conclusions:**

The prevalent segregation of the c.212-37insC polymorphism in children with CaA advocates a possible functional *FGF23* role in the predisposition to higher serum levels of FGF23 and potential occurrence of any coronary artery abnormalities in KS.

## 

Kawasaki syndrome (KS), a systemic panvasculitis of unknown origin affecting all medium/small-sized vessels, typically occurring in early childhood, bears an outstanding risk of cardiac abnormalities (CaA), ranging from asymptomatic coronary artery ectasia to giant aneurysms [1]: these might occur in 15-25% of untreated cases during the subacute phase of KS, and an increasing body of evidence supports a widespread vascular dysfunction in KS with risk of atherogenesis during adulthood of these patients [2].

The discovery of fibroblast growth factor 23 (FGF23), a bone-derived hormone that regulates systemic phosphate homeostasis and vitamin D metabolism via FGF-receptor 1 (FGFr1)/cofactor Klotho, which is included in a previously unrecognized hormonal bone-parathyroid-kidney axis [3], has uncovered new implications for skeletal fragility, renal disease-associated morbidity, and even cardiovascular pathology [4].

In the present study we have investigated the genetic variation in the *FGF23* gene in a cohort of children with a confirmed diagnosis of KS and its association with serum FGF23 levels and development of CaA.

## Patients and methods

Our study population included 84 consecutive children of Italian ancestry, 62 males and 22 females, with a mean age of 40.8 months, who fulfilled the American Heart Association criteria for the diagnosis of KS. Every patient was treated with intravenous immunoglobulin (IVIG, 2 g/kg over 10–12 hours within the 10^th^ day since disease onset) and aspirin (50–80 mg/kg/day in 4 doses during the acute phase). All patients were immunoglobulin-responsive, and no repeated cycles of IVIG were required. No additional therapies were adopted. Fever duration (in days) was recorded for each patient. Endocrinological and chronic renal diseases, which can be associated with inappropriately high serum FGF23 levels or phosphate wasting and impaired bone mineralization, were excluded in all patients. Each patient underwent evaluation of serum FGF23 levels (expressed in pg/ml and measured by an ELISA assay, Immunotopics Inc. San Clemente, CA, USA; lower limit of detection: 1.0 pg/ml) at the time of inclusion in the study (i.e. when diagnosis of KS was formulated), in combination with the assessment of different laboratory data: erythrosedimentation rate (ESR), C-reactive protein (CRP), haemoglobin (Hb), platelet count, fibrinogen, total cholesterol, HDL cholesterol, LDL cholesterol, and triglycerids. All patients underwent 2D-echocardiogram at admission and routinely, if coronary artery involvement was absent. Twenty-four/84 KS patients (28.6%) developed CaA in terms of coronary artery complications (14 patients had coronary aneurysms and/or dilatations of whatever dimensions) and pericardial effusion (in 10 patients): all these children were more closely controlled by 2D-echocardiograms, performed always by the same pediatric cardiologist.

### Fibroblast growth factor 23 gene evaluation

Both ethical approval from the Ethics Committee of the University of Florence and a formal informed consent by relatives or tutors of each child were obtained. Patients’ DNA was extracted from peripheral blood mononuclear cells. The three *FGF23* exons, including the intron-exon boundary regions, were PCR-amplified and analyzed on ABI Prism 3100 Genetic Analyzer (Applied Biosystems, Foster City, CA). Primer sequences were as follows: exon 1for GGGGTCTTTGCACTTTCTTTC; exon 1rev GGTTGGATTAGCCCTCCAGT; exon 2for ATCAATCCAGGGAGGTTTCA; exon 2rev GGAAACAGGTCACCAGGGTA; exon 3for AGGAGGAGCTGGGGAGTG and exon 3rev GACCTGGTCCTTGGGAAGA. PCR was performed with 1.5 mM magnesium chloride, 0.2 mM deoxynucleotide triphosphates, 0.2 μM of each primer, 1 U of Taq polymerase and 100 ng of genomic DNA as template. The obtained sequences were compared with the wild type reference sequence of the gene, published on Genbank Database (NT-009759). *FGF23* gene analysis revealed the polymorphism c.212-37insC in intron 1, characterized by a C insertion in the intronic region between −36 and −37 nucleotide (rs3832879: NM_020638.2:c.212-37_212-36insC), in a subset of KS patients. Subjects without this *FGF23* polymorphism were indicated as wild type.

### Statistical analysis

Univariate statistical analysis of categorical parameters was performed by the Pearson’s Chi-square test or Fisher’s exact test as appropriate. Parametric variables were assessed by Student’s t-test for unpaired data. The comparison of age of onset between the two groups was performed by Mann–Whitney U test. Variables significantly associated with CaA at the univariate analysis were entered into a logistic regression model to identify independent predictors of disease. Values were expressed as total count [% ratio] for categorical variables, means ± SD for parametric variables and value [95% Confidence Interval] for Odds ratio. Age of onset was expressed as median [quartiles]. A *p* value of 0.05 or less was considered as significant.

## Results

Twenty-eight patients with KS carried the *FGF23* c.212-37insC polymorphism (33.3%). We found that male sex was significantly associated with the polymorphism (91.7% *vs* 61.7%, *p* = 0.03). Patients presenting the *FGF23* polymorphism had also significantly higher serum FGF23 levels (41.4 pg/ml ± 53.9 *vs* 10.7 pg/ml ± 13.5, *p* < 0.01). In addition, the *FGF23* polymorphism was significantly associated with CaA: indeed, patients presenting with coronary dilatations or aneurysms were significantly more represented in the subgroup with *FGF23* polymorphism, compared to wild type *FGF23* children (29.2% *vs* 11.7%, *p* = 0.05). The number of patients with only coronary aneurysms (3 patients) was too small to obtain statistical significance in a separate subgroup analysis. Neither pericardial effusion, nor the overall signs of cardiac involvement (in the whole) were significantly associated with the *FGF23* polymorphism. No demographical, clinical or laboratory data were found to be associated with the *FGF23* polymorphism.

At the multivariate analysis, FGF23 polymorphism was independently associated with both CaA and high serum FGF23 levels (respectively Odds ratios: 4.3 [1.1-16.2], *p* = 0.03 and 1.05 [1.0-1.1], *p* = 0.01). Male sex, that showed a strong univariate association with the *FGF23* polymorphism, did not reach a statistical significance at the multivariate analysis: however, since the *FGF23* polymorphism was mostly observed in males (22 out of 24, 91.7%), we probably have not sufficient data to determine its real impact in the male sex (see Table 1).

**Table 1:** **Univariate statistical analysis with demographic/laboratory variables and cardiac abnormalities in comparison with the *FGF23 *** **gene polymorphism in the cohort of 84 children with Kawasaki syndrome recruited for this study**

**Variable**	***FGF23 *** **wild gene**	***FGF23 *** **polymorphism**	***p *** **value**
Sex (M/F)	40/20	22/2	0.03
	(66.7%-33.3%)	(91.7%-8.3%)	
Age of onset (in months)	24.5 [13.2-49.5]	36 [16.7-58.0]	0.39
Fever duration (in days)	8.7 ± 4.0	9.3 ± 3.8	0.75
***Laboratory data***			
Serum FGF23	10.7 ± 13.5	41.4 ± 53.9	0.01
ESR at onset (mm/1 h)	83.3 ± 22.7	83.5 ± 19.4	0.44
CRP at onset (mg/L)	14.0 ± 15.8	17.3 ± 16.9	0.52
Hb at onset (g/L)	10.6 ± 1.1	10.2 ± 0.9	0.48
Platelet count at onset (×10^9^/L)	450.8 ± 142.1	433.0 ± 124.1	0.86
Fibrinogen at onset (mg/dl)	672.33 ± 216.6	732.6 ± 162.3	0.27
Total cholesterol (mg/dl)	162.2 ± 34.0	171.8 ± 22.3	0.13
HDL cholesterol (mg/dl)	39.5 ± 14.6	39.6 ± 12.5	0.59
LDL cholesterol (mg/dl)	103.7 ± 39.7	105.6 ± 29.6	0.44
Triglycerides (mg/dl)	86.5 ± 58.1	89.7 ± 76.4	0.65
***Cardiac abnormalities***			
Coronary dilatations	6/54	7/17	0.03
(present/absent)	(10.0%/90.0%)	(29.2%/70.8%)	
Coronary aneurysms	2/58	1/23	0.64
(present/absent)	(3.3%/96.7%)	(4.2%/95.8%)	
Coronary aneurysms or dilatations	7/53	7/17	0.05
(present/absent)	(11.7%/88.3%)	(29.2%/70.8%)	
Pericardial effusion	7/53	4/20	0.54
(present/absent)	(11.7%/88.3%)	(16.7%/83.3%)	
Any cardiac involvement	14/46	10/14	0.09
(present/absent)	(23.3%/76.7%)	(41.7%/58.3%)	

All parametric data are expressed as mean ± SD. Age of onset is expressed as median [quartiles]. Categorical variables are expressed as raw numbers (%).

## Discussion

Endothelium dysfunction is an established trigger for cardiovascular accidents in the general population and is a major pathogenetic mechanism involved in KS [5]. FGF23, which is part of hormonal bone-parathyroid-kidney axis, modulated by parathyroid hormone, 1,25(OH)2-vitamin D, diet and serum phosphorus levels, exerts its bioactivity on selected target-tissues interacting with its receptor FGFr, largely diffuse in bone tissue and different endothelia, in the presence of Klotho co-factor [6]. Elevated serum levels of FGF23 have been demonstrated in children with rickets and diminished bone mineral density, but also a direct FGF23 effect has been hypothesized on the heart [7,8]. In a previous study we have observed that intact serum FGF23 levels in children with KS were significantly higher when compared with healthy controls, in particular those displaying CaA, revealing the potential role of FGF23 as a marker suggestive of cardiac complications [9]. Unfortunately there are no other published studies on the association between vasculitides in childhood and FGF23, and clinical trials are needed to determine whether FGF23 is a modifiable risk factor.

In this study we have investigated the genetic variation in the *FGF23* gene in a cohort of 84 children with KS and its correlation with serum FGF23 levels and development of CaA: we have found a significant correlation between the *FGF23* rs3832879 variant (which can be found in 15.6% of the population; http://www.ncbi.nlm.nih.gov/snp/?term=rs3832879) and both serum FGF23 levels and CaA, including both coronary artery dilatations and aneurysms. In particular, patients with the *FGF23* polymorphism had higher serum FGF23 levels (*p* < 0.01) and developed more frequently coronary artery dilatations or aneurysms (*p* = 0.05). Finally, the multivariate analysis showed that both CaA and serum FGF23 were independently associated with the *FGF23* polymorphism (respectively *p* = 0.03 and *p* = 0.01).

In summary, coronary artery dilatations were demonstrated in 29.2% of children bearing the *FGF23* polymorphism and only in 10% of wild type *FGF23* patients: this striking difference might support the functional role that this polymorphism could display on the susceptibility to CaA, although the details of the interaction of *FGF23* gene with other genetic systems remain unclear at present.

From these preliminary results, which need to be replicated on larger samples of patients, the segregation of the *FGF23* polymorphism in children with KS who developed CaA suggests its potential contribution to the development of cardiovascular complications. Whether this genotype may act in synergy with other genes or specific environmental factors remains to be elucidated in further studies.

## Abbreviations

FGF23: Fibroblast Growth Factor 23; KS: Kawasaki syndrome; CaA: Cardiac abnormalities; IVIG: Intravenous immunoglobulins; ESR: Erythrocyte sedimentation rate; CRP: C-reactive protein; Hb: Haemoglobin.

## Competing interests

The authors declare that they have no competing interests.

## Authors’ contributions

FF (submitting author), LM, MMC, and MLB primarily created the protocol of the study; FF and DR drafted the manuscript and revised it based on all co-authors’ suggestions; GT and MC made the statistical analysis of the study; FF and GL performed the genetical assays in patients recruited in the study. FF and DR equally contributed to the overall production of this study and manuscript. All authors read and approved the final manuscript.
